# Risk factors for BPD and relevant nursing models of care to improve outcomes: a narrative review

**DOI:** 10.3389/fped.2026.1734364

**Published:** 2026-04-24

**Authors:** Fangfang Lei, Jie Xiang, Xuemei Guo, Xia Li, Yaohua Wu, Dapeng Chen, Yanling Hu

**Affiliations:** 1 Department of Obstetric Nursing, West China Second University Hospital, Sichuan University, Key Laboratory of Birth Defects and Related Diseases of Women and Children (Sichuan University), Ministry of Education, Chengdu, Sichuan, China; 2 Department of Neonatal intensive care units, West China Second University Hospital, Sichuan University, Key Laboratory of Birth Defects and Related Diseases of Women and Children (Sichuan University), Ministry of Education, Chengdu, Sichuan, China; 3 Department of Pediatrics, West China Second University Hospital, Sichuan University, Key Laboratory of Birth Defects and Related Diseases of Women and Children (Sichuan University), Ministry of Education, Chengdu, Sichuan, China

**Keywords:** Bronchopulmonary dysplasia (BPD), chronic lung disease of prematurity (CLD), clinical outcomes, nursing care, preterm infants, risk factors

## Abstract

Bronchopulmonary dysplasia (BPD) is the most common chronic respiratory disease in premature infants, posing a serious threat to their lives and affecting their long-term quality of life. The smaller the gestational age at birth, the higher the incidence of BPD in preterm infants. The authors conducted a narrative review of the high-risk factors for development of BPD and outline the major nursing interventions used in the care of such infants.

## 1 Introduction

Bronchopulmonary dysplasia (BPD) was first described by Northway and colleagues in 1967 as a chronic lung injury caused by barotrauma and oxygen toxicity in preterm infants requiring mechanical ventilation (1). According to the latest criteria updated by the National Institute of Child Health and Human Development, the definition and severity of BPD are now primarily graded based on the specific model of respiratory support required at 36 weeks’ postmenstrual age (2, 3). Despite significant advances in preterm infant care over the past few decades, the prevalence of this condition remains high (4). In fact, advancements in perinatal medicine and neonatal resuscitation techniques have enabled more premature newborns to survive, which has led to a rise in the incidence of BPD. BPD is closely associated with increased mortality and poor long-term outcomes in preterm infants. Specifically, BPD increases the risk of premature death, and survivors are frequently hospitalized before the age of two due to respiratory diseases (5). As adults, they may experience decreased physical abilities, obstructive airway diseases, emphysema, pulmonary hypertension, and frequent respiratory infections (6). Research has shown that differences in medical and nursing care affect the quality of care, mortality rates, and long-term outcomes for preterm infants with BPD (7). This review explores high-risk factors for development of BPD and nursing interventions for its optimal management such that outcomes can be improved for preterm infants.

## 2 Risk factors and clinical determinants of BPD

The development of Bronchopulmonary Dysplasia (BPD) is driven by a complex interplay of multiple factors.

### 2.1 Core risk factors: gestational Age and birth weight

Gestational age and birth weight at delivery are the most critical predictors of BPD. The International Neonatal Outcome Collaborative reported an overall BPD incidence of 22% among premature infants (8). Data from the China Neonatal Collaborative Network further highlight a sharp inverse correlation between gestational age and BPD risk: the incidence is 29.2% for gestational age <32 weeks, 24.4% for 28 weeks to 31 weeks +6 days, 51.9% for 26 weeks to 27 weeks +6 days, and surges to 74.2% for infants <26 weeks (9). Additionally, male infants exhibit a higher susceptibility to BPD compared to females (10).

### 2.2 Antenatal and perinatal factors

BPD risk is influenced by genetic predisposition and maternal conditions, including hypertension, smoking history, chorioamnionitis, intrauterine hypoxia, and placental insufficiency (11, 12). Delivery circumstances also play a pivotal role; perinatal asphyxia, coupled with high oxygen concentrations and excessive pressure during resuscitation, can cause acute lung injury that progresses to BPD (5, 13).

### 2.3 Postnatal physiological vulnerabilities and management

The inherent physiological immaturity of preterm infants underpins BPD progression: 1) underdeveloped lungs with surfactant deficiency; 2) immature renal function, making it difficult to maintain fluid stability, which leads to high positive cumulative fluid balance (CFB) and pulmonary interstitial edema; 3) inadequate caloric intake, which delays physical growth and impairs lung repair, thereby prolonging mechanical ventilation (14). Furthermore, anemia and red blood cell transfusions (received by 80% of VLBW infants) are independently associated with increased BPD rates (15). Infections trigger inflammatory cascades and the release of reactive oxygen species, leading to cell necrosis and are also linked to worsening BPD (16). Notably, the high rate and prolonged use of broad-spectrum antibiotics in NICUs may also contribute to the risk of developing BPD (17).

### 2.4 Evolving perspectives on patent ductus arteriosus (PDA)

While various postnatal morbidities influence the development and severity of BPD, the specific role of PDA remains a subject of intense debate. Historically, a hemodynamically significant PDA was considered a primary contributor to BPD. However, despite observational studies suggesting a link between prolonged PDA exposure and increased BPD rates, recent randomized controlled trials have not consistently demonstrated that pharmacological or surgical closure reduces the overall risk of BPD (18). Notably, the BeNeDuctus trial established that expectant management is non-inferior to early active treatment regarding the composite outcome of BPD or death (19). Current evidence suggests that early targeted management of PDA might offer respiratory benefits primarily in a specific subpopulation of extremely preterm infants (<26 weeks’ gestation) (20). Nevertheless, meta-analyses indicate that the certainty of evidence supporting early intervention to prevent BPD in this demographic remains low (21). Consequently, clinical strategies have increasingly shifted from routine closure toward individualized hemodynamic monitoring and expectant management.

## 3 Nursing model

### 3.1 Family participatory nursing model

Family-Centered Participatory Care (FI Care) is a transitional care model that integrates Developmental Care with Family-Centered Care (22). Under the guidance of medical staff, this model empowers parents to become the primary caregivers and participate in routine nursing operations during their premature infant's stay in the NICU. The interventions of this model mainly consist of four aspects: parental education, peer support, staff training, and environmental support. The development of FI Care model has become more mature internationally. In China, scholar Mingyan Hei (23) initiated the first domestic clinical research on FI Care in 2014. Currently, FI Care clinical studies are being carried out in medical institutions in cities such as Beijing, Shanghai and other places. Parents in the NICU providing non-medical care, engaging in enhanced skin-to-skin contact, and offering comfort improves the rate of breastfeeding in premature infants, which is protective against BPD (24). It also helps shorten the transition period from total parenteral nutrition to full enteral feeding (25). Additionally, it can positively improve the psychological health of the parents. The separation of mother and baby increases the anxiety of parents, but the FI Care model allows parents to actively participate in the care of their premature infant in the NICU, boosting their confidence and happiness. Furthermore, learning caregiving skills and emergency knowledge alleviates their uncertainty and confusion about caregiving after discharge, and it can also reduce the rate of readmission after discharge (26). For infants with BPD, who have long hospital stays, the FI Care model is beneficial for both the premature infant and the family. However, this model of care has not traditionally been practiced in China and requires cultural adjustments, as well as investments to modify the ward environment. This includes setting up specialized family-participation wards, teaching rooms, family lounges, and additional support staff. Furthermore, professional training for the FI Care team is needed. This model may also conflict with China's traditional “postpartum confinement” practices, necessitating ongoing exploration and change management.

### 3.2 Bundles of care

Bundles of care refer to the integration of a series of evidence-based treatments and improvement measures to address specific issues e.g, difficult-to-manage clinical conditions (27). The initial clinical application of bundled care interventions was in the prevention of ventilator-associated pneumonia (VAP) (28). Research applying bundled care interventions to preterm infants has shown that they can significantly reduce mechanical ventilation time, shorten hospital stays, and decrease the incidence of lung infections (29). Another study found that bundled care could reduce the incidence of VAP in preterm infants, improve their prognosis, and identify the main pathogens responsible for VAP, enabling targeted antibiotic use and preventing the overuse of broad-spectrum antibiotics (30). In clinical settings, nurses’ lack of experience may affect the rate of unplanned extubation in intubated patients, but bundled care interventions can significantly reduce this rate (31). Bundled care facilitates the translation of clinical evidence and guidelines into nursing practice and if implemented using effective improvement methodology can contribute to improved clinical care quality and outcomes.

### 3.3 Kangaroo care

Kangaroo Mother Care (KMC) is a recognized neonatal intervention consisting of three core components: kangaroo position, kangaroo nutrition, and kangaroo discharge (32). The kangaroo position, also referred to as skin-to-skin care, involves continuous skin contact between the parent and the newborn, resembling the way a kangaroo carries its young. Kangaroo nutrition refers to exclusive breastfeeding, where, although other methods may be initially used to meet the infant's needs, the goal is to ultimately achieve exclusive breastfeeding. Kangaroo discharge emphasizes early discharge from the hospital to minimize the negative effects of the hospital environment on the infant. In 2003, the World Health Organization (WHO) issued the “Clinical Practice Guidelines for Kangaroo Mother Care”, and in 2015, WHO recommended that KMC be routinely applied to all newborns weighing less than 2000g (33). WHO also recommended that KMC be implemented once the premature infant's condition stabilizes. One of the most common complications in preterm infants is apnea of prematurity (AOP). The use of KMC has been shown to be a protective factor against AOP, reducing the frequency of AOP episodes and shortening the duration of oxygen therapy. When applying KMC, the preterm infant's position is changed from lying flat to a 60–90° upright prone position on the mother or father's chest. This position enhances comfort, reduces oxygen consumption, and improves the infant's oxygenation capacity (34). Skin-to-skin contact with the mother or father provides warmth and a sense of security for the infant, reducing stress, and when the infant is calm, the frequency of AOP episodes decreases. KMC has demonstrated significant benefits for preterm infants, including improvements in neurodevelopment, physical growth, exclusive breastfeeding, long-term quality of life, and a reduction in parental anxiety and depression (35, 36). Research on KMC in China began relatively late, with most studies focusing on the kangaroo position stage, and there is limited research on extremely preterm and very low birth weight infants. Furthermore, KMC may conflict with traditional Chinese postnatal practices, which emphasizes maternal rest after childbirth. This creates challenges in balancing the mother's recovery with the optimization of KMC. To overcome these cultural barriers, current clinical practice and research in China are increasingly exploring the father's role in KMC as an equally important alternative (37).

### 3.4 Continuity of care

Continuity of care refers to a series of nursing activities designed to ensure that patients receive coordinated and continuous health services when transitioning between different healthcare settings or levels of care, preventing or reducing the deterioration of health conditions for high-risk patients (38). The definition emphasizes the transfer, connection, coordination, and consistency of nursing tasks. It is commonly used in the care of elderly patients, those with chronic diseases, and cancer patients. In recent years, it has also gained increasing attention in the field of neonatal care, particularly in the NICU for premature infants. The Kenner Transition Model is a well-established continuity of care model for preterm infants abroad. It divides the home care challenges and concerns of parents of preterm infants into five categories: knowledge needs, stress and coping, grief, social interactions, and parent-child role development (39–41). The Transition Questionnaire has been developed to evaluate the continuity of care challenges faced by parents of preterm infants. However, this questionnaire has not yet been cross-culturally adapted or applied to neonatal continuity of care in China.

Preterm infants with BPD require long-term oxygen therapy support and continue to need home oxygen therapy, sustained nutritional support, and care after discharge. Caregivers need to be equipped with relevant skills such as nasogastric feeding, medication administration, sputum clearance, and prevention of aspiration. The United States was one of the first countries to implement home-based continuity of care, providing post-discharge care for NICU infants. The care includes assessments of the home environment, feeding guidance, and management of the infant's medication regimen (42). In China, continuity of care has already been implemented in postpartum maternal and infant health care. Although it has helped reduce readmission rates and improve both short- and long-term quality of life for infants, it has not yet addressed the management of BPD in preterm infants. In China, there is still no unified standard for continuity of care in preterm infants. The models and duration of continuity care vary from hospital to hospital, and there is no established health education evaluation standard for continuity of care in preterm infants outside of the hospital. Furthermore, effective connections between hospital and community care have not yet been established.

### 3.5 Refined nursing care

Refined nursing care is a detailed and structured management approach that has been applied in neonatal settings, such as for the prevention of hospital-acquired infections and the promotion of breastfeeding. It emphasizes a patient-centered philosophy, aiming to improve patient comfort, support recovery, and potentially shorten hospitalization time. Research indicates that refined nursing care facilitates earlier initiation of breastfeeding and increases the proportion of exclusive breastfeeding in preterm infants compared to standard care, which helps reduce the risk of BPD in preterm infants (40, 41). Studies have also suggested that replacing the traditional bag-valve-mask with a T-piece resuscitator for positive pressure ventilation, along with personalized adjustments of ventilator parameters and oxygen concentration based on gestational age, helps reduce the risk of BPD (43, 44). Due to their compromised immunity, preterm infants should be closely monitored for infection. The correct and standardized use of isolation gowns, as well as strict hand hygiene before and after handling preterm infants, are essential (45). Following the implementation of refined nursing management, the incidence of ventilator-associated pneumonia, central line-associated bloodstream infections, and diaper dermatitis in preterm infants has been shown to decrease (46). The core focus of this model is ensuring accuracy, efficiency, and continuous coordination at each stage of care. Establishing a specialised nursing team looking after preterm infants with BPD can improve the theoretical knowledge and practical skills of neonatal nurses, thereby enhancing the overall quality of neonatal care. Furthermore, specialised nursing involves multidisciplinary collaboration, including medical, nursing, and allied health professional teams, as this integrated approach can further improve clinical outcomes for preterm infants.

## 4 Nursing measures

While the aforementioned nursing models provide broad, organizational frameworks for NICU care, the specific prevention and management of BPD rely on targeted clinical interventions. The overall treatment goals for BPD in premature infants are to maintain precise fluid balance to improve lung function, provide adequate nutrition to promote lung repair, and ensure physical growth and neurodevelopment of the infant (47). Based on these goals, the nursing measures for premature infants with BPD can be categorized into four main areas: airway management, nutritional support, infection control, and fluid management and balance.

### 4.1 Airway care

Premature infants are at an increased risk of mortality if they experience hypoxia within the first five minutes after birth. It is essential to conduct immediate oxygen saturation monitoring and adjust oxygen delivery to ensure that the saturation level are appropriate (48). Unnecessary tracheal intubation and mechanical ventilation is best avoided to prevent secondary ventilator-associated injuries. However, if mechanical ventilation is unavoidable, it is recommended to use Volume Targeted Ventilation and High Frequency Oscillatory Ventilation to improve respiratory support (44). These methods help provide more precise ventilation volumes and reduce the risk of lung injury. Airway secretions can increase airway resistance and affect gas exchange in the lungs. When providing non-invasive or invasive ventilatory support for premature infants, it is essential to ensure that their airways are clear. Guidelines recommend performing suctioning as needed, with the suctioning duration kept as brief as possible, and using the “nesting position” to reduce pain and stress during suctioning (49). Additionally, the guidelines suggest that prone positioning can improve ventilation function in BPD premature infants and shorten the duration of mechanical ventilation (49). However, while therapeutic prone positioning is beneficial under continuous clinical monitoring, it is advised that premature infants avoid unmonitored sleeping in the prone position to reduce the risk of sudden infant death syndrome (50). Premature infants born at a gestational age of less than 29 weeks often experience one or more episodes of unplanned extubation during mechanical ventilation. Moreover, re-intubation following unplanned extubation and prolonged duration of mechanical ventilation can increase the risk of developing BPD in these infants (51).These aspects need to manage within the bundles of care developed for improving outcomes.

### 4.2 Nutritional support

Premature infants born at lower gestational ages and experiencing slower growth after birth have a higher risk of developing BPD (52). Clinically, it is essential to strictly adhere to nutritional guidelines for premature infants, providing sufficient nutrition that includes protein, energy, and micronutrients.

Breast milk has a protective effect on BPD in premature infants, which can reduce the incidence of necrotizing enterocolitis and late-onset sepsis in these infants, as well as prevent ventilator-associated pneumonia (53). Donor milk also has some beneficial effect, and it is recommended to use donor breast milk to provide the necessary nutritional support when a mother's breast milk is insufficient after delivery (54). Measures to improve the feeding process of premature infants primarily include colostrum oral care, non-nutritional sucking, and oral motor interventions. Both non-nutritive sucking and oral motor interventions can shorten the time for premature infants to achieve full oral feeding, and enhance breastfeeding rates, thereby reducing the length of hospital stays (55). Colostrum oral care can help protect against respiratory viral invasion and prevent the occurrence of ventilator-associated pneumonia (53). By promoting oral feeding through nursing interventions, we can reduce feeding intolerance rates and ensure adequate nutritional support.

### 4.3 Infection control

Macrolide antibiotics, such as azithromycin, erythromycin, and clarithromycin, have been investigated for BPD prevention due to the known association between Ureaplasma and Mycoplasma colonization and the development of the disease. While early animal models (56) and some smaller clinical studies (57) suggested that azithromycin might attenuate pulmonary inflammation and reduce BPD incidence, recent robust clinical evidence does not support this approach. Notably, the Azithromycin Therapy for Chronic Lung Disease of Prematurity trial—a large-scale, multicenter, randomized placebo-controlled study—demonstrated that a 10-day course of intravenous azithromycin did not improve survival without physiologically defined moderate or severe BPD in extremely preterm infants, regardless of their Ureaplasma colonization status (56). Consequently, the routine prophylactic use of macrolides to prevent BPD is not recommended in current clinical practice.

### 4.4 Fluid management and balance

After birth, premature infants require adequate nutritional support to facilitate somatic growth and lung maturation. While fluid overload can exacerbate pulmonary edema due to immature renal and respiratory function, strict fluid restriction may compromise caloric and nutrient intake. Consequently, current clinical guidance emphasizes the need for monitoring of CFB rather than solely restricting fluid intake volume. Evidence suggests that a higher positive CFB within the first 10 days of life is associated with an increased incidence of BPD (58, 59). Therefore, strategies aiming to optimize cumulative fluid balance without impeding nutritional delivery are prioritized. Diuretics, such as furosemide, are utilized to manage acute fluid retention and improve pulmonary mechanics (60). However, due to potential adverse effects, including electrolyte disturbances, nephrotoxicity, and ototoxicity, their prolonged use is generally not recommended.

## 5 Strengths and limitations

The main strength of this review is its focus on aspects of non-medical care especially nursing models and within the cultural context in China. Several limitations must be noted. First, since this is a narrative review, the lack of a systematic methodology may introduce selection bias, limiting the strength of the evidence. Secondly, the high degree of heterogeneity in study designs and sample sizes across the discussed nursing models affects the generalizability of the findings. Most existing studies on these nursing care models and interventions focus on short-term outcomes, with limited data on long-term neurodevelopmental and respiratory health. Finally, while mention is made of practices pertinent to China, this has not been investigated or discussed in a methodical way.

## 6 Conclusion

BPD remains a critical chronic respiratory complication in preterm infants, with its incidence inversely correlated with gestational age. This review underscores that optimal clinical outcomes require a synergistic approach that integrates core clinical interventions—such as airway management, nutritional support, infection control, and fluid balance management—within evidence-based nursing frameworks. While emerging nursing models, including FI Care, KMC, and Bundled Care, have demonstrated significant potential in improving respiratory outcomes and reducing hospital stays, their clinical application is currently hindered by a lack of standardized protocols and unified implementation pathways across different healthcare settings.

## References

[1] Northway WH Jr. Rosan RC Porter DY. Pulmonary disease following respirator therapy of hyaline-membrane disease. Bronchopulmonary dysplasia. N Engl J Med. (1967) 276(7):357–68. 10.1056/nejm196702162760701 5334613

[2] Higgins RD Jobe AH Koso-Thomas M Bancalari E Viscardi RM Hartert TV Bronchopulmonary dysplasia: executive summary of a workshop. J Pediatr. (2018) 197:300–8. 10.1016/j.jpeds.2018.01.043 29551318 PMC5970962

[3] Jensen EA Dysart K Gantz MG McDonald S Bamat NA Keszler M The diagnosis of bronchopulmonary dysplasia in very preterm infants. An evidence-based approach. Am J Respir Crit Care Med. (2019) 200(6):751–9. 10.1164/rccm.201812-2348OC 30995069 PMC6775872

[4] Sahni M Mowes AK. Bronchopulmonary dysplasia. In StatPearls. Tampa: StatPearls Publishing (2026) Copyright © 2026, StatPearls Publishing LLC. 30969701

[5] Thébaud B Goss KN Laughon M Whitsett JA Abman SH Steinhorn RH Bronchopulmonary dysplasia. Nat Rev Dis Primers. (2019) 5(1):78. 10.1038/s41572-019-0127-7 31727986 PMC6986462

[6] Malleske DT Chorna O Maitre NL. Pulmonary sequelae and functional limitations in children and adults with bronchopulmonary dysplasia. Paediatr Respir Rev. (2018) 26:55–9. 10.1016/j.prrv.2017.07.002 29031795

[7] Zhu Z Yuan L Wang J Li Q Yang C Gao X Mortality and morbidity of infants born extremely preterm at tertiary medical centers in China from 2010 to 2019. JAMA Netw Open. (2021) 4(5):e219382. 10.1001/jamanetworkopen.2021.9382 33974055 PMC8114138

[8] Shah PS Lui K Sjörs G Mirea L Reichman B Adams M Neonatal outcomes of very low birth weight and very preterm neonates: an international comparison. J Pediatr. (2016) 177:144–152.e146. 10.1016/j.jpeds.2016.04.083 27233521

[9] Cao Y Jiang S Sun J Hei M Wang L Zhang H Assessment of neonatal intensive care unit practices, morbidity, and mortality among very preterm infants in China. JAMA Netw Open. (2021) 4(8):e2118904. 10.1001/jamanetworkopen.2021.18904 34338792 PMC8329742

[10] Laube M Thome UH. Y it matters-sex differences in fetal lung development. Biomolecules. (2022) 12(3). 10.3390/biom12030437 PMC8946560 35327629

[11] Hadchouel A Decobert F Besmond C Delacourt C. Exome sequencing of extreme phenotypes in bronchopulmonary dysplasia. Eur J Pediatr. (2020) 179(4):579–86. 10.1007/s00431-019-03535-0 31848748

[12] Morrow LA Wagner BD Ingram DA Poindexter BB Schibler K Cotten CM Antenatal determinants of bronchopulmonary dysplasia and late respiratory disease in preterm infants. Am J Respir Crit Care Med. (2017) 196(3):364–74. 10.1164/rccm.201612-2414OC 28249118 PMC5549867

[13] Hillman NH Kallapur SG Jobe AH. Physiology of transition from intrauterine to extrauterine life. Clin Perinatol. (2012) 39(4):769–83. 10.1016/j.clp.2012.09.009 23164177 PMC3504352

[14] Malikiwi AI Lee YM Davies-Tuck M Wong FY. Postnatal nutritional deficit is an independent predictor of bronchopulmonary dysplasia among extremely premature infants born at or less than 28 weeks gestation. Early Hum Dev. (2019) 131:29–35. 10.1016/j.earlhumdev.2019.02.005 30825742

[15] Tao Y Han X Guo WL. Predictors of bronchopulmonary dysplasia in 625 neonates with respiratory distress syndrome. J Trop Pediatr. (2022) 68(3):fmac037. 10.1093/tropej/fmac037 35595255 PMC9122647

[16] Kalikkot Thekkeveedu R Guaman MC Shivanna B. Bronchopulmonary dysplasia: a review of pathogenesis and pathophysiology. Respir Med. (2017) 132:170–7. 10.1016/j.rmed.2017.10.014 29229093 PMC5729938

[17] Jiang S Zhang L Yan W Li S Han J Zhou Q Antibiotic use in neonatal intensive care units in China: a multicenter cohort study. J Pediatr. (2021) 239:136–142.e134. 10.1016/j.jpeds.2021.08.067 34461063

[18] Sankar M-N Bhombal S Benitz W-E. PDA: to treat or not to treat. Struct Congenit Heart Dis. (2019) 14(1):46–51. Available online at: http://www.techscience.com/schd/v14n1/38741 10.1111/chd.12708 30811796

[19] Hundscheid T Onland W Kooi EMW Vijlbrief DC de Vries WB Dijkman KP Expectant management or early ibuprofen for patent ductus arteriosus. N Engl J Med. (2023) 388(11):980–90. 10.1056/NEJMoa2207418 36477458

[20] Clyman RI Couto J Murphy GM. Patent ductus arteriosus: are current neonatal treatment options better or worse than no treatment at all? Semin Perinatol. (2012) 36(2):123–9. 10.1053/j.semperi.2011.09.022 22414883 PMC3305915

[21] Mitra S Florez ID Tamayo ME Mbuagbaw L Vanniyasingam T Veroniki AA Association of placebo, indomethacin, ibuprofen, and Acetaminophen with closure of hemodynamically significant patent ductus arteriosus in preterm infants: a systematic review and meta-analysis. JAMA. (2018) 319(12):1221–38. 10.1001/jama.2018.1896 29584842 PMC5885871

[22] O'Brien K Bracht M Macdonell K McBride T Robson K O'Leary L A pilot cohort analytic study of family integrated care in a Canadian neonatal intensive care unit. BMC Pregnancy Childbirth. (2013) 13(Suppl 1):S12. 10.1186/1471-2393-13-s1-s12 23445639 PMC3561192

[23] Hei M Gao X Li Y Gao X Li Z Xia S Family integrated care for preterm infants in China: a cluster randomized controlled trial. J Pediatr. (2021) 228:36–43.e32. 10.1016/j.jpeds.2020.09.006 32898578

[24] Meek JY Noble L. Policy statement: breastfeeding and the use of human milk. Pediatrics. (2022) 150(1). 10.1542/peds.2022-057988 35921640

[25] Chen S Shen H Jin Q Zhou L Feng L. Family-centered care in the neonatal intensive care unit: a meta-analysis and systematic review of outcomes for preterm infants. Transl Pediatr. (2025) 14(1):14–24. 10.21037/tp-24-373 39944875 PMC11811580

[26] Taneja S Bahl S Mazumder S Martines J Bhandari N Bhan MK. Impact on inequities in health indicators: effect of implementing the integrated management of neonatal and childhood illness programme in Haryana, India. J Glob Health. (2015) 5(1):010401. 10.7189/jogh.05.010401 25674350 PMC4306296

[27] Lachman P Yuen S. Using care bundles to prevent infection in neonatal and paediatric ICUs. Curr Opin Infect Dis. (2009) 22(3):224–8. 10.1097/QCO.0b013e3283297b68 19369867

[28] Al-Tawfiq JA Abed MS. Decreasing ventilator-associated pneumonia in adult intensive care units using the institute for healthcare improvement bundle. Am J Infect Control. (2010) 38(7):552–6. 10.1016/j.ajic.2010.01.008 20400203

[29] Huskins WC. Quality improvement interventions to prevent healthcare-associated infections in neonates and children. Curr Opin Pediatr. (2012) 24(1):103–12. 10.1097/MOP.0b013e32834ebdc3 22189394

[30] Zhou Q Lee SK Jiang SY Chen C Kamaluddeen M Hu XJ Efficacy of an infection control program in reducing ventilator-associated pneumonia in a Chinese neonatal intensive care unit. Am J Infect Control. (2013) 41(11):1059–64. 10.1016/j.ajic.2013.06.007 24041863

[31] da Silva PSL Reis ME Farah D Andrade TRM Fonseca MCM. Care bundles to reduce unplanned extubation in critically ill children: a systematic review, critical appraisal and meta-analysis. Arch Dis Child. (2022) 107(3):271–6. 10.1136/archdischild-2021-321996 34284999

[32] Charpak N Ruiz-Peláez JG Figueroa de CZ Charpak Y. Kangaroo mother versus traditional care for newborn infants </=2000 grams: a randomized, controlled trial. Pediatrics. (1997) 100(4):682–8. 10.1542/peds.100.4.682 9310525

[33] Chan GJ Valsangkar B Kajeepeta S Boundy EO Wall S. What is kangaroo mother care? Systematic review of the literature. J Glob Health. (2016) 6(1):010701. 10.7189/jogh.06.010701 27231546 PMC4871067

[34] Özdel D Sarı HY. Effects of the prone position and kangaroo care on gastric residual volume, vital signs and comfort in preterm infants. Jpn J Nurs Sci. (2020) 17(1):e12287. 10.1111/jjns.12287 31642602

[35] Vogl JL Dunne EC Liu C Bradley A Rwei A Lonergan EK Kangaroo father care: a pilot feasibility study of physiologic, biologic, and psychosocial measures to capture the effects of father-infant and mother-infant skin-to-skin contact in the neonatal intensive care unit. Dev Psychobiol. (2021) 63(5):1521–33. 10.1002/dev.22100 33521969

[36] Wang Y Zhao T Zhang Y Li S Cong X. Positive effects of kangaroo mother care on long-term breastfeeding rates, growth, and neurodevelopment in preterm infants. Breastfeed Med. (2021) 16(4):282–91. 10.1089/bfm.2020.0358 33533688

[37] Deng Q Li Q Wang H Sun H Xu X. Early father-infant skin-to-skin contact and its effect on the neurodevelopmental outcomes of moderately preterm infants in China: study protocol for a randomized controlled trial. Trials. (2018) 19(1):701. 10.1186/s13063-018-3060-2 30577818 PMC6303962

[38] Shortell SM Rundall TG Hsu J. Improving patient care by linking evidence-based medicine and evidence-based management. JAMA. (2007) 298(6):673–6. 10.1001/jama.298.6.673 17684190

[39] Flandermeyer A Kenner C Spaite ME Hostiuck J. Transition from hospital to home. Part II. Neonatal Netw. (1992) 11(5):62–3. 1495468

[40] Fonseca LT Senna DC Silveira RC Procianoy RS. Association between breast milk and bronchopulmonary dysplasia: a single center observational study. Am J Perinatol. (2017) 34(3):264–9. 10.1055/s-0036-1586503 27487230

[41] Waldron MK. NICU Parents of black preterm infants: application of the kenner transition model. Adv Neonatal Care. (2022) 22(6):550–9. 10.1097/anc.0000000000000980 35588065

[42] Hummel P Cronin J. Home care of the high-risk infant. Adv Neonatal Care. (2004) 4(6):354–64. 10.1016/j.adnc.2004.09.011 15609257

[43] Aziz K Lee HC Escobedo MB Hoover AV Kamath-Rayne BD Kapadia VS Part 5: neonatal resuscitation: 2020 American Heart Association guidelines for cardiopulmonary resuscitation and emergency cardiovascular care. Circulation. (2020) 142(16_suppl_2):S524–50. 10.1161/cir.0000000000000902 33081528

[44] Sweet DG Carnielli VP Greisen G Hallman M Klebermass-Schrehof K Ozek E European Consensus guidelines on the management of respiratory distress syndrome: 2022 update. Neonatology. (2023) 120(1):3–23. 10.1159/000528914 36863329 PMC10064400

[45] Legeay C Bourigault C Lepelletier D Zahar JR. Prevention of healthcare-associated infections in neonates: room for improvement. J Hosp Infect. (2015) 89(4):319–23. 10.1016/j.jhin.2015.02.003 25748794 PMC7172434

[46] Schulman J Stricof R Stevens TP Horgan M Gase K Holzman IR Statewide NICU central-line-associated bloodstream infection rates decline after bundles and checklists. Pediatrics. (2011) 127(3):436–44. 10.1542/peds.2010-2873 21339265

[47] Hongping XIA ZY. Phenotypic characteristics and treatment strategies of severe bronchopulmonary dysplasia. J Clin Pediatr. (2022) 40(6):401–6. 10.12372/jcp.2022.22e0605

[48] Gilfillan M Bhandari A Bhandari V. Diagnosis and management of bronchopulmonary dysplasia. Br Med J. (2021) 375:n1974. 10.1136/bmj.n1974 34670756

[49] The Subspecialty Group of Neonatology, the Society of Pediatrics, Chinese Medical Association, & the Editorial Board. (2020). Expert consensus on clinical management of premature infants with bronchopulmonary dysplasia. Chin J Pediatr, 58(7):521–8. 10.3760/cma.j.cn112140-20200317-00254 32392950

[50] Oyen N Markestad T Skaerven R Irgens LM Helweg-Larsen K Alm B Combined effects of sleeping position and prenatal risk factors in sudden infant death syndrome: the nordic epidemiological SIDS study. Pediatrics. (1997) 100(4):613–21. 10.1542/peds.100.4.613 9310514

[51] Le Blanc G Jabbour E Patel S Kazantseva O Zeid M Olivier F Organizational risk factors and clinical impacts of unplanned extubation in the neonatal intensive care unit. J Pediatr. (2022) 249:14–21.e15. 10.1016/j.jpeds.2022.06.012 35714965

[52] Xu RZ Sun B Zhao NC. Association between early parenteral nutrition and the development of bronchopulmonary dysplasia in preterm infants. Zhongguo Dang Dai Er Ke Za Zhi. (2023) 25(4):362–7. 10.7499/j.issn.1008-8830.2210128 (早期肠外营养与早产儿支气管肺发育不良发生的关系.) 37073840 PMC10120333

[53] Rodriguez NA Meier PP Groer MW Zeller JM Engstrom JL Fogg L. A pilot study to determine the safety and feasibility of oropharyngeal administration of own mother's colostrum to extremely low-birth-weight infants. Adv Neonatal Care. (2010) 10(4):206–12. 10.1097/ANC.0b013e3181e94133 20697221 PMC2924875

[54] Villamor-Martínez E Pierro M Cavallaro G Mosca F Kramer BW Villamor E. Donor human milk protects against bronchopulmonary dysplasia: a systematic review and meta-analysis. Nutrients. (2018) 10(2):238. 10.3390/nu10020238 29461479 PMC5852814

[55] Chen D Yang Z Chen C Wang P. Effect of oral motor intervention on oral feeding in preterm infants: a systematic review and meta-analysis. Am J Speech Lang Pathol. (2021) 30(5):2318–28. 10.1044/2021_ajslp-20-00322 34314255

[56] Lowe J Gillespie D Aboklaish A Lau TMM Consoli C Babu M Azithromycin therapy for prevention of chronic lung disease of prematurity (AZTEC): a multicentre, double-blind, randomised, placebo-controlled trial. Lancet Respir Med. (2024) 12(8):608–18. 10.1016/S2213-2600(24)00079-1 38679042

[57] Smith C Egunsola O Choonara I Kotecha S Jacqz-Aigrain E Sammons H. Use and safety of azithromycin in neonates: a systematic review. BMJ Open. (2015) 5(12):e008194. 10.1136/bmjopen-2015-008194 26656010 PMC4679913

[58] Sharma R Bhandari V. Fluid balance in early postnatal life: should we keep the babies dry to prevent bronchopulmonary dysplasia? Pediatr Res. (2021) 90(2):240–1. 10.1038/s41390-021-01589-1 34035427

[59] Soullane S Patel S Claveau M Wazneh L Sant'Anna G Beltempo M. Fluid status in the first 10 days of life and death/bronchopulmonary dysplasia among preterm infants. Pediatr Res. (2021) 90(2):353–8. 10.1038/s41390-021-01485-8 33824447

[60] Segar JL. Neonatal diuretic therapy: furosemide, thiazides, and spironolactone. Clin Perinatol. (2012) 39(1):209–20. 10.1016/j.clp.2011.12.007 22341547

